# Evidence of docosahexaenoic acid deficiency in maple syrup urine disease: insights from plasma long-chain polyunsaturated fatty acid status

**DOI:** 10.1038/s41430-026-01774-7

**Published:** 2026-06-12

**Authors:** Tanyel Zubarioglu, Özgür Kasapçopur, Sedanur Akça-Yeşil, Esra İşat, Mehmet Şerif Cansever, Ertuğrul Kıykım, Çiğdem Aktuğlu-Zeybek

**Affiliations:** 1 Department of Pediatric Nutrition and Metabolism, İstanbul University-Cerrahpaşa, Cerrahpaşa Medical Faculty, İstanbul, Turkey; 2 Department of Pediatric Nutrition, İstanbul University-Cerrahpaşa, Institute of Graduate Studies, İstanbul, Turkey; 3 Department of Pediatrics, İstanbul University-Cerrahpaşa, Cerrahpaşa Medical Faculty, İstanbul, Turkey; 4 Nutrition and Dietetics Unit, İstanbul University-Cerrahpaşa, Cerrahpaşa Medical Faculty, İstanbul, Turkey; 5 Research Laboratory of Metabolism, İstanbul-University-Cerrahpaşa, Cerrahpaşa Medical Faculty, İstanbul, Turkey; 6 Department of Medical Laboratory Techniques, Istanbul University-Cerrahpaşa, The Vocational School of Health Services, İstanbul, Turkey

**Keywords:** Nutrition, Paediatrics, Metabolic disorders

## Abstract

**Background:**

Maple syrup urine disease (MSUD) is an inherited metabolic disorder requiring protein restriction, often limiting intake of animal-derived foods. This raises concerns about long-chain polyunsaturated fatty acid (LC-PUFA) status. The primary aim of this study is to evaluate plasma n–3 and n–6 fatty acid levels in MSUD patients.

**Methods:**

This single-center, cross-sectional study included 16 MSUD patients and 22 unaffected siblings sharing a similar household and environmental background. Dietary intake was recorded and plasma fatty acid profiles were analyzed.

**Results:**

Dietary assessments revealed significantly lower intakes of total fat and cholesterol (both *p* < 0.001) and n-3 PUFAs (*p* = 0.036) in MSUD patients, with alpha-linolenic acid (ALA) intake as the only individual LC-PUFA significantly reduced (*p* = 0.048). Plasma analysis showed significantly lower docosahexaenoic acid (DHA) levels in patients despite similar dietary DHA intake (*p* < 0.001), while arachidonic acid and mead acid were significantly elevated (both *p* < 0.001). Although plasma DHA concentrations showed a moderate positive correlation with dietary ALA intake (r = 0.516, *p* = 0.041), regression analysis showed that neither dietary ALA intake (B = 0.005, *p* = 0.584) nor the dietary n-6/n-3 PUFA ratio (B = −0.552, *p* = 0.706) independently predicted plasma DHA levels. In the MSUD group, plasma DHA levels were positively associated with dietary leucine intake (r = 0.635, *p* = 0.008) and plasma isoleucine concentrations (r = 0.524, *p* = 0.037).

**Conclusion:**

Our findings provide evidence of DHA deficiency in MSUD patients, which may result from both inadequate dietary intake and changes in n-3 PUFA metabolism, highlighting the need to investigate additional contributing mechanisms.

## Introduction

Maple syrup urine disease (MSUD) is an autosomal recessive inherited metabolic disease (IMD) caused by biallelic pathogenic variants in the *BCKDHA, BCKDHB* or *DBT* genes encoding the catalytic subunits and components of the enzyme branched-chain α-ketoacid dehydrogenase (BCKD). This mitochondrial enzyme complex decarboxylates α-ketoacid derivatives (BCKAs) of the branched-chain amino acids (BCAAs) -leucine, isoleucine and valine and its deficiency results in the accumulation of BCAAs and their corresponding BCKAs [1, 2]. The clinical phenotype of MSUD is classified into four types -classic, intermittent, intermediate and thiamine-responsive-based on residual enzyme activity, age of onset, symptom severity and responsiveness to thiamine treatment. Among these, classic MSUD is considered one of the most volatile and life-threatening inherited metabolic diseases. It typically presents in the neonatal period with profound metabolic encephalopathy, severe cerebral edema, and acute elevations of leucine and its corresponding ketoacid, α-ketoisocaproic acid. However, individuals with intermediate or intermittent forms of MSUD are also at risk of experiencing acute metabolic decompensation and encephalopathy during periods of catabolic stress, despite having milder baseline presentations. Acute metabolic crises can be life-threatening, while chronic manifestations such as intellectual disability, developmental delay and motor deficits may occur in individuals with inadequately treated or poorly controlled MSUD [1, 2]. Therefore, lifelong medical nutrition therapy is the cornerstone of chronic MSUD management. This involves a protein-restricted diet that limits dietary intake of BCAAs to levels that allow plasma BCAA concentrations to remain within target treatment ranges. The primary goals of this approach are to prevent catabolism, support anabolism and promote normal growth and neurodevelopment [2, 3].

Long-chain polyunsaturated fatty acids (LC-PUFAs) are structurally diverse fatty acids containing more than 18 carbon atoms and multiple double bonds. They are vital components of cellular membranes in nearly all tissues, contributing to membrane fluidity, permeability and receptor functionality. LC-PUFAs influence cellular signaling by modulating the number and affinity of membrane-bound receptors and serve as precursors for a wide range of bioactive lipid mediators [4]. The most biologically relevant LC-PUFAs include arachidonic acid (AA; C20:4n–6), docosahexaenoic acid (DHA; C22:6n–3) and eicosapentaenoic acid (EPA; C20:5n–3). These fatty acids are synthesized from the essential fatty acid precursors linoleic acid (LA; C18:2n–6) and alpha-linolenic acid (ALA; C18:3n–3), which cannot be produced endogenously in humans due to the lack of specific desaturase enzymes and therefore must be obtained through the diet [4]. The conversion of LA and ALA into LC-PUFAs occurs through a series of alternating desaturation and elongation steps. This process is primarily regulated by Δ6-desaturase (D6D) and Δ5-desaturase (D5D), encoded by the *FADS2* and FADS1 genes, respectively, as well as elongase enzymes such as *ELOVL2* and *ELOVL5*. Enzyme activity in this pathway is influenced by both genetic and nutritional factors [4–6].

Dietary intake is the principal source of LC-PUFAs, particularly for their preformed long-chain metabolites. While vegetable oils are rich in essential fatty acids like ALA and LA, the long-chain n-6 and n-3 fatty acids such as AA, EPA and DHA are primarily obtained from animal-derived foods. Foods from terrestrial animals are generally rich in n-6 fatty acids, whereas marine fish and seafood provide significant amounts of n-3 LC-PUFAs, especially EPA and DHA [7–10]. Given the inability of mammals to synthesize essential fatty acids and the limited efficiency of ALA-to-DHA conversion, adequate dietary intake of preformed LC-PUFAs is crucial, particularly for supporting neural development, retinal function, and cardiovascular health [4]. In many IMDs, especially those requiring lifelong dietary management, patients face restrictions on a wide range of natural protein sources, including both animal- and plant-derived proteins, resulting in a severely limited intake of natural proteins. This dietary pattern raises important concerns about whether synthetic diets provide adequate amounts of essential fatty acids, to what extent endogenous biosynthetic pathways can compensate for the absence of preformed DHA, and whether such limitations necessitate targeted LC-PUFA supplementation. These concerns have been widely studied, particularly in phenylketonuria (PKU), where fatty acid status has been evaluated across multiple pediatric and adult cohorts [11–16]. However, in other IMDs, existing studies are extremely limited and often based on small sample sizes [17–19]. In MSUD, chronic dietary management typically includes supplementation with vitamins, minerals and essential fatty acids [3]. However, scientific evidence supporting LC-PUFA supplementation is limited [20, 21], and its inclusion in treatment protocols is not yet standardized, highlighting the need for further investigation.

The primary objective of this study is to evaluate the plasma concentrations of selected n–3 and n–6 fatty acids in patients with MSUD compared to healthy controls.

## Materials and methods

### Study design and participants

This study was designed as a single-center, cross-sectional study conducted between October 2024 and July 2025. A total of 38 participants, including 16 MSUD patients and 22 healthy controls were enrolled.

The patient group included MSUD patients who met the following inclusion criteria:

- A biochemical and/or molecularly confirmed diagnosis of MSUD
- Receiving protein-restricted medical nutrition therapy
- Good adherence to treatment and follow-up confirmed by the treating physician.

The healthy control group consisted of unaffected siblings from the same household to minimize differences in socioeconomic status and environmental exposure, rather than differences in dietary composition.

Participants who received targeted omega-3 fatty acid supplementation (DHA- or EPA-containing products) within the three months before enrollment were excluded to avoid direct interference with plasma fatty acid measurements. This criterion did not include standard medical nutrition products used as part of routine MSUD dietary management. Individuals with diagnosed dyslipidemia, chronic gastrointestinal disorders affecting fat absorption, severe malnutrition or other comorbid conditions known to influence lipid metabolism were also excluded. MSUD patients who had undergone liver transplantation were excluded due to altered amino acid and lipid metabolism after transplantation. Healthy control participants following vegetarian or vegan diets were excluded to minimize dietary heterogeneity related to fatty acid intake and to ensure a more comparable dietary background between groups.

All adult participants provided written informed consent before enrollment. For participants under 18 years of age, written informed consent was obtained from parents, and age-appropriate assent was obtained from the minors when applicable. For participants with limited decision-making capacity, written informed consent was obtained from their legal guardians. Ethical approval was taken by the local ethics committee (approval number: E-83045809804.01-1150436).

### Dietary assessment

Anthropometric measurements, including height (cm), body weight (kg) and body mass index (BMI, kg/m²), were obtained from all participants. Body weight was measured with a calibrated digital scale (Desi B5 model; precision ±0.1 kg). Height or length was assessed using age- and mobility-appropriate methods: standing height was measured with a stadiometer (Desi B5 model) in ambulatory participants, while recumbent length was measured in non-ambulatory participants using a non-flexible measuring tape following standardized length measurement techniques. All anthropometric measurements were performed by the same, trained personnel according to a standardized protocol. Corresponding z-scores were calculated using national reference data and local growth charts. Participants were classified as pediatric (<18 years) or adult (≥18 years) based on the conventional age cutoff of 18 years, allowing for the appropriate use of BMI z-scores in children and absolute BMI values in adults. Dietary intake was assessed using a self-reported 3-day food record that included two weekdays and one weekend day. Participants and/or caregivers were instructed to record all food and beverage consumption in detail, including portion sizes and brand names where applicable. For MSUD patients, detailed notes were taken regarding the use of BCAA-free amino acid mixtures, including product names, quantities consumed and frequency of intake.

Nutrient analysis was performed using the Nutrition Information System (BeBiS), version 8.2. The mean daily intake of energy (DCI, kcal/day), carbohydrates (% of DCI), total fat (% of DCI), total protein (g/kg/day), natural protein (g/kg/day), leucine (mg/day), n-3 PUFAs (mg/day), n-6 PUFAs (mg/day), saturated fat (% of DCI), cholesterol (mg/day), DHA (mg/day), EPA (mg/day), LA (mg/day) and ALA (mg/day) was calculated. Additionally, the dietary n-6/n-3 ratio was calculated for each participant to evaluate the balance of PUFA in the diet.

### Clinical and laboratory assessment

All patients and their siblings were invited for a scheduled clinical visit during a metabolically stable period. For each individual, a single venous blood sample was collected after 8 h of fasting. None of the samples were obtained during an acute metabolic crisis. At the time of sample collection, all patients underwent a standardized clinical evaluation to rule out signs of metabolic decompensation. This evaluation included assessment of neurological status (altered level of consciousness, seizures or ataxia) and laboratory parameters indicative of a metabolic crisis (urinary ketone bodies and urine dinitrophenylhydrazine test).

During the same visit, venous blood samples were collected simultaneously for lipid profiling [triglycerides (TG), total cholesterol (TC), low-density lipoprotein cholesterol (LDL-C), and high-density lipoprotein cholesterol (HDL-C), all expressed in mg/dL], plasma quantitative amino acid analysis (µmol/L), and LC-PUFA measurement from all participants. A portion of each blood sample was centrifuged at 3000 × *g* for 10 min at 4 °C, and the resulting plasma was stored at –80 °C until further analysis. Plasma samples were subsequently analyzed to determine the concentrations of selected LC-PUFAs.

### Procedures for fatty acid analysis

#### Analytical method and reagents

Quantitative analysis of n-3 and n-6 PUFAs in plasma samples was performed using the Jasem Omega Fatty Acids Gas Chromatography-Mass Spectrometry (GC-MS) Analysis Kit (Jasem Co., Turkey). This validated commercial kit enables rapid and accurate determination of esterified fatty acids by GC-MS. The following reagents and components provided in the kit were used according to the manufacturer’s instructions: ready-to-use derivatization reagents, calibrator standard mixture, internal standard solution and quality control serum.

After derivatization, the method quantifies methyl ester derivatives of serum fatty acids using an Agilent 6980/5973 GC-MS system. The analytical workflow was optimized using the kit’s integrated protocol, which includes esterification, extraction, and GC-MS measurement steps. Each run was completed in ~9.3 min, following the manufacturer’s validated procedure (Jasem Method). Chromatographic outputs were evaluated with Jasem HP ChemStation software. Fatty acids were identified by both retention time and mass spectral data, and their concentrations were calculated using internal standard methodology. Final results were reported in mg/L based on GC-MS quantification.

#### Calibration, validation and analysis

For each analyte, a six-point calibration curve was constructed. The linear regression coefficients (R²) ranged from 0.9950 to 0.9998, indicating excellent linearity across the analytical range. Method validation was performed according to the performance parameters provided by the manufacturer. These parameters, consistent with the Jasem validation data, are summarized in Supplementary Material S1.

The following fatty acids were analyzed in plasma samples: 18:3n–3 (alpha-linolenic acid; ALA), 20:5n–3 (eicosapentaenoic acid; EPA), 22:6n–3 (docosahexaenoic acid; DHA), 18:2n–6 (linoleic acid; LA), 18:3n–6 (gamma-linolenic acid; GLA), 20:4n–6 (arachidonic acid; AA) and 20:3 n-9 (Mead acid -5,8,11-eicosatrienoic acid ;MA).

### Statistical analysis

Statistical analyses were performed using the Statistical Package for Social Sciences version 22.0 (SPSS Inc., Chicago, IL, USA). Descriptive analyses were presented as mean ± standard deviation or median (25th; 75th percentiles), according to data distribution. Categorical variables were presented as numbers (percentages). The normality of data distribution was evaluated with the Shapiro–Wilk test. Categorical variables were analyzed using the Chi-square test. The independent samples *t*-test was used for normally distributed quantitative variables, and the Mann–Whitney U test was used for comparisons of non-normally distributed data between two groups. Associations between dietary intake variables and plasma fatty acid levels were initially examined using Spearman’s rank correlation coefficient, given the non-normal distribution of the data and the small sample size. For correlations involving dichotomous variables, such as sex, point-biserial correlation analysis was used. To further assess the combined predictive value of dietary variables on plasma fatty acid status, multiple linear regression analyses were conducted using the enter method. Regression model fit was evaluated with the F-test and the coefficient of determination (R²). The contribution of individual predictors was assessed using unstandardized and standardized beta coefficients, corresponding *t* values, and *p* values. A *p*-value < 0.05 was considered statistically significant.

## Results

### Demographic, clinical and genetic features of the patients

**Fig. 1:**
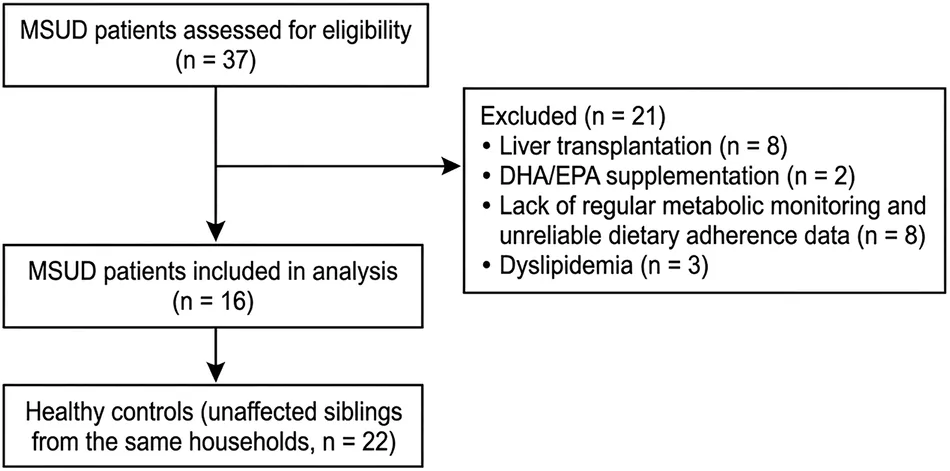
Participant flow diagram of the study population. A total of 37 MSUD patients were assessed for eligibility, with 21 excluded based on predefined criteria (liver transplantation, *n* = 8; DHA/EPA supplementation, *n* = 2; unreliable metabolic and dietary data, *n* = 8; dyslipidemia, *n* = 3), resulting in 16 MSUD patients included in the final analysis. The control group consisted of unaffected siblings recruited from the same households.

A total of 37 MSUD patients were initially assessed for eligibility. Of these, 21 were excluded for the following reasons: liver transplantation (*n* = 8), use of DHA/EPA-containing fatty acid supplementation (*n* = 2), lack of regular metabolic monitoring and unreliable dietary adherence data (*n* = 8) and dyslipidemia (*n* = 3). A total of 16 patients with MSUD (42.1%) and 22 healthy controls (57.9%) were included in the study. The participant flow diagram is shown in Fig. 1. Eight (50%) of the MSUD patients were female and the median age was 11.59 (8.57; 18.94) years. There were no statistically significant differences between the patient and control groups in age (*p* = 0.246) or sex distribution (*p* = 0.578). Demographic characteristics and genotypic data related to MSUD, are presented in Supplementary Material S2.

At the time of plasma fatty acid profiling, the mean plasma leucine concentration in patients was 197.08 ± 110.24 µmol/L and none of the patients exhibited clinical signs of acute metabolic decompensation. In addition, review of longitudinal metabolic monitoring showed that the mean monthly plasma leucine levels over the preceding year were 91.14 ± 44.11 µmol/L, supporting good dietary adherence in the patient group.

Among the patient group, 93.8% (*n* = 15) were fed orally, while one patient received nutrition via percutaneous endoscopic gastrostomy. Regarding food texture, one patient was fed a liquid formula and another consumed pureed food. The remaining 87.5% consumed food in all textures and forms. All patients were receiving age-appropriate BCAA-free amino acid mixtures as part of standard dietary management for MSUD. In addition, seven patients (43.7%) were using powdered low-protein milk replacement products to increase daily caloric intake and meet individual energy requirements. These products are not intended as fatty acid supplements but may contain varying amounts of fatty acids. The macronutrient composition of the medical foods used in the study, including their fatty acid content and patient-level usage, is provided in Supplementary Material S3.

### Anthropometric and dietary assessment

In the pediatric subgroup, no significant differences were observed in body weight z score or BMI z score between patients with MSUD (*n* = 11) and healthy controls (*n* = 14). However, height z score was significantly lower in the patient group (*p* = 0.002). In the adult subgroup, although BMI values were higher in patients, this difference did not reach statistical significance. Data on the anthropometric characteristics of both groups are presented in Table 1.

**Table 1:** Anthropometric characteristics of the participants.

Pediatric cohort	MSUD patients (*n* = 11)^a^	Healthy controls (*n* = 14)^a^	*p*-value^b^
Age (years)	9.31 (7.49;13.37)	11.98 (9.34;13.50)	0.267
Sex			
*Male*	7 (63.6)	5 (35.7)	0.165^c^
*Female*	4 (36.4)	9 (64.3)	0.165^c^
Weight z score	−0.32 (−0.77;1.53)	0.50 (−0.63; 1.16)	0.647
Height z score	−1.78 (−3.08; −0.11)	0.33 (−0.24; 0.70)	**0.002**
BMI z score	0.73 (−0.08; 2.26)	0.40 (−0.71; 1.19)	0.403
**Adult cohort**	**Study group** **(*n*** = **5)**^**a**^	**Healthy controls** **(*n*** = **8)**^**a**^	***p*-value**^**b**^
Age (years)	20.69 (18.66;24.52)	24.14 (22.31;27.66)	0.171
Sex			
*Male*	1 (20)	4 (50)	0.565^d^
*Female*	4 (80)	4 (50)	
Weight (kg)	69.9(49.6;101.7)	62.25(54.07;71.12)	0.833
Height (cm)	162(152.5;165)	165(159.5;171.5)	0.222
BMI (kg/m^2^)	26.6 (21.35; 37.55)	22.20 (19.32; 24.95)	0.171

*BMI* Body mass index.

^a^Median (Q1;Q3), *n* (%).

^b^Mann–Whitney U.

^c^Pearson’s Chi-squared test.

^d^Fisher’s exact test.

Bold values indicate statistically significant *p*-values (*p* < 0.05).

When evaluating dietary fat intake, MSUD patients had significantly lower fat consumption compared to controls (*p* < 0.001). Cholesterol intake was also markedly reduced in the patient group (*p* < 0.001), which is expected given the typically low intake of natural protein and animal-derived foods in MSUD dietary management. Dietary assessment revealed that oleic acid intake was significantly lower in MSUD patients than in controls (*p* = 0.010).

Notably, n-3 PUFA intake was significantly lower in the patient group (*p* = 0.036). Although the control group showed a trend toward higher n-6 PUFA intake, this difference was not statistically significant. When LC-PUFA intake was analyzed separately, there were no statistically significant differences between groups in LA, EPA and DHA intakes. However, intake of ALA was significantly lower in the patient group (*p* = 0.048).

Dietary intake parameters, including total energy, macronutrients and fatty acid profiles for both groups, are summarized in Table 2.

**Table 2:** Comparison of daily energy, macronutrient, and fatty acid intake between patients with MSUD and healthy controls.

	MSUD patients (*n* = 16)^a^	Healthy controls (*n* = 22)^a^	*p*-value^c^
Total energy (kcal)	1632.50 (1478.25;1804)	1457.50(1352.75;1654.75)	**0.031**
CHO (% of DCI)	51.12 ± 8.98^b^	44.18 ± 11^b^	**0.046**^**d**^
Fat (% of DCI)	27.12 ± 11.96^b^	41.72 ± 9.56^b^	**<0.001**^**d**^
Total protein (gr/kg)	2.28(2.08;2.53)	1.09(0.81;1.35)	**<0.001**
Natural protein (gr/kg)	0.24(0.13;0.36)	1.09(0.81;1.35)	**<0.001**
Leucine (mg)	512.63 ± 295.59^b^	4040.59 ± 1368.78^b^	**<0.001**^**d**^
Valine (mg)	511.04(319.15;1207.45)	2527.70(1931.12;3387.82)	**<0.001**^**d**^
Isoleucine (mg)	855.50(370.25;1473.50)	2293.50(1563.75;3092.25)	**<0.001**^**d**^
Saturated fat (% of DCI)	10.48 (6.18;15.16)	14.04 (6.53;18.50)	0.341
Cholesterol (mg)	0.55 (0.22;22.37)	321 (157;438)	**<0.001**
n-3 PUFA (mg)	450 (400;1000)	1200 (575;1650)	**0.036**
n-6 PUFA (mg)	2950 (2700;8625)	8950 (3675;15050)	0.084
Oleic acid (mg)	17125 ± 5239.91^b^	23740.90 ± 8650.33^b^	**0.010**
EPA (mg)	200 (125;375)	200 (100;350)	0.715
DHA (mg)	0 (0;100)	50 (0;125)	0.404
LA (mg)	2950 (2625;9550)	9350 (3750;15225)	0.073
ALA (mg)	400 (400;850)	950 (500;1450)	**0.048**
n-6 PUFA /n-3 PUFA ratio	6.86 ± 3.14^b^	8.96 ± 4.59^b^	0.125^2^

*CHO* carbohydrate, *DCI* daily caloric intake, *n-3* omega-3, *n-6* omega-6, *PUFA* polyunsaturated fatty acid, *EPA* eicosapentaenoic acid, *DHA* docosahexaenoic acid, *LA* linoleic acid, *ALA* alpha-linolenic acid.

^a^Median (Q1;Q3).

^b^Mean ± SD.

^c^Mann–Whitney U.

^d^Independent sample *t* test.

Bold values indicate statistically significant *p*-values (*p* < 0.05).

### Plasma fatty acid profile assessment

Regarding plasma n-3 and n-6 fatty acid composition, AA levels were significantly higher in the MSUD patients compared to healthy controls (*p* < 0.001). No significant differences were found between groups for GLA, LA ALA or EPA. However, DHA levels were significantly lower in the patient group (*p* < 0.001). Levels of plasma MA, an n-9 PUFA, were also significantly higher in the patient group (*p* < 0.001). However, the median MA/AA (triene/tetraene) ratio was 0.34 (0.33;0.36) in MSUD patients and 0.35 (0.34;0.35) in the control group, with no significant difference between the groups (*p* = 0.298).

**Fig. 2:**
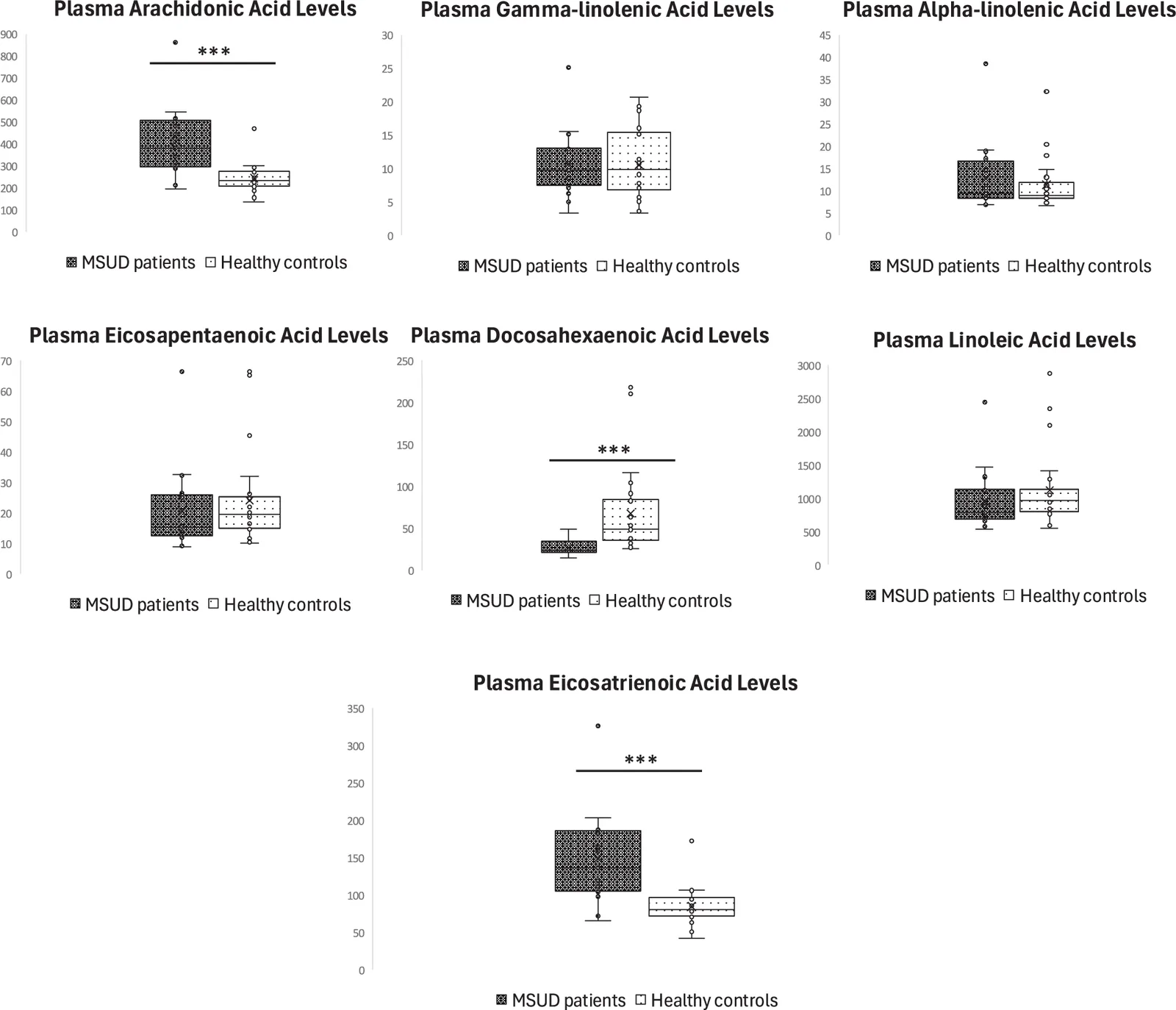
Plasma long-chain polyunsaturated fatty acid profiles in patients with MSUD and healthy controls. The figure shows group comparisons of plasma n-6 and n-3 long-chain polyunsaturated fatty acids, including linoleic acid, gamma-linolenic acid, arachidonic acid, alpha-linolenic acid, eicosapentaenoic acid, docosahexaenoic acid, and mead acid (eicosatrienoic acid, 20:3n-9). Patients with MSUD had significantly higher levels of arachidonic acid and mead acid, and significantly lower levels of docosahexaenoic acid compared with healthy controls, indicating altered fatty acid metabolism (***p < 0.001).

Simultaneous assessment of standard lipid parameters, including HDL, LDL, triglycerides and TC, showed no statistically significant differences between the groups. Detailed data on plasma lipid and fatty acid profiles are presented in Table 3 and Fig. 2.

**Table 3:** Data on the plasma lipid and fatty acid profile of patients with MSUD and healthy controls.

	MSUD patients (*n* = 16)^a^	Healthy controls (*n* = 22)^a^	*p*-value^b^
*Lipid profile(mg/dL)*			
Total cholesterol	132 (124;146.75)	141 (126.75;163.75)	0.312
HDL-C	49 (44.25;56.07)	49 (42;57.25)	0.849
LDL-C	71 (51;86.5)	78 (71;92.25)	0.095
Triglyceride	81.5 (60.5;127.5)	64 (50.5;106.25)	0.108
*Fatty acid profile(mg/L)*			
Arachidonic acid	379.22 (298.06;508.33)	234.43 (209.24;272.98)	**<0.001**
Gamma-linolenic acid	9.72 (7.49;13.01)	9.83 (6.76;15.36)	0.895
Alpha-linolenic acid	9.30 (8.40;16.56)	9.04 (8.32;11.87)	0.849
Eicosapentaenoic acid	15.04 (12.54;25.93)	19.47 (14.7;25.11)	0.258
Docosahexaenoic acid	22.4 (20.4;33.9)	48.05 (34.65;84.23)	**<0.001**
Linoleic acid	787.60 (685.88;1128.50)	960.65 (799.71;1126.69)	0.191
Eicosatrienoic acid	136.56 (105.51;186.30)	80.55 (71.8;96.30)	**<0.001**

*HDL-C* high-density lipoprotein cholesterol, *LDL-C* low-density lipoprotein cholesterol.

^a^Median (Q1;Q3).

^b^Mann–Whitney U.

Bold values indicate statistically significant *p*-values (*p* < 0.05).

### Branched-chain amino acids and their relationship with plasma DHA levels

Reflecting the dietary restrictions required for MSUD management, dietary intake of BCAAs was significantly lower in MSUD patients than in controls (all *p* < 0.001). Data on the dietary BCAAs intake are presented in Table 2.

At the time of fatty acid sampling, median plasma BCAA levels were significantly higher in MSUD patients than in controls. In the MSUD group, median plasma concentrations were 203.15 µmol/L (108; 268.25) for leucine, 484 µmol/L (337; 705.75) for valine, and 269.50 µmol/L (166.87; 483.75) for isoleucine. In the control group, the corresponding values were 116.80 µmol/L (108.80; 143.75), 269.50 µmol/L (166.87; 483.75), and 88.95 µmol/L (83.32; 94.07), respectively. These differences were statistically significant for all three amino acids (*p* = 0.026 for leucine, *p* = 0.001 for valine and *p* < 0.001 for isoleucine).

Given that plasma DHA levels were significantly lower in the MSUD group, correlation analyses were conducted within this group to explore potential associations with dietary and metabolic variables. Dietary leucine intake showed a significant positive association with plasma DHA levels (r = 0.635, *p* = 0.008). Among plasma BCAA levels, plasma isoleucine was also positively correlated with plasma DHA concentrations (r = 0.524, *p* = 0.037), while no significant associations were observed for plasma leucine or valine levels (*p* > 0.05 for both). No significant correlations were found between plasma DHA levels and dietary valine or isoleucine intake (*p* > 0.05 for both).

### Assessment of factors associated with plasma DHA status in MSUD patients

To investigate potential determinants of reduced DHA levels observed in the patient group, both bivariate correlation and multivariate regression analyses were conducted.

In the correlation analyses, no significant associations were observed between plasma DHA concentrations and dietary n-6/n-3 PUFA ratio or age. A borderline association was observed between plasma DHA levels and dietary n-3 PUFA intake (r = 0.497, *p* = 0.050). The only statistically significant association was a medium positive correlation between plasma DHA concentrations and dietary ALA intake (r = 0.516, *p* = 0.041). Using point-biserial correlation analysis, no significant association was observed between plasma DHA concentrations and sex (r = 0.271, *p* = 0.310). Detailed statistical data from the correlation analyses are provided in Supplementary Material S4.

To further investigate the predictive value of selected dietary variables on plasma fatty acid status, a multiple linear regression analysis was conducted with plasma DHA concentration as the dependent variable. Correlation analyses revealed a high correlation between dietary ALA intake and total dietary n-3 PUFA intake, indicating potential multicollinearity between these variables. To prevent model instability, only dietary ALA intake was included in the regression model along with the dietary n-6/n-3 PUFA ratio. In the MSUD group, the regression model was not statistically significant (F(2,13) = 0.237, *p* = 0.792) and explained only 3.5% of the variance in plasma DHA concentrations (R² = 0.035). Neither dietary ALA intake (B = 0.005, *p* = 0.584) nor the dietary n-6/n-3 PUFA ratio (B = −0.552, *p* = 0.706) was a significant predictor of plasma DHA levels. Detailed multiple linear regression analysis data on the associations between dietary fatty acid intake and plasma DHA concentrations are summarized in Table 4.

**Table 4:** Multiple linear regression analysis of dietary predictors of plasma DHA concentrations in patients with MSUD.

Independent variables	Unstandardized β	Standard error	Standardized β	*t*-value	*p*-value
Dietary ALA intake (mg/day)	0.005	0.008	0.153	0.561	0.584
Dietary n-6/n-3 PUFA ratio	−0.552	1.433	−0.105	−0.385	0.706

Regression coefficients are derived from multiple linear regression models using the enter method, with plasma DHA concentration as the dependent variable and dietary fatty acid variables as independent predictors. Overall model statistics are presented in the Results section. No additional covariates were included in the models to avoid overfitting due to the limited sample size and the absence of significant associations in preliminary analyses.

*PUFA* polyunsaturated fatty acid, *DHA* docosahexaenoic acid, *ALA* alpha-linolenic acid.

## Discussion

This study provides a comprehensive evaluation of dietary intake and plasma long-chain PUFA status, highlighting evidence of DHA deficiency in MSUD patients. Dietary assessments showed significantly lower total fat, cholesterol and n-3 PUFA intake in the patient group, consistent with the protein-restricted, low-animal product diets characteristic of MSUD management. Although DHA intake did not differ significantly between groups, plasma DHA concentrations were markedly reduced in MSUD patients, suggesting a possible disruption in endogenous conversion pathways. In contrast, AA and MA – a potential marker of essential fatty acid deficiency when interpreted relative to other fatty acid indices – were significantly elevated in the patient group. Exploratory analyses also suggested some associations between BCAAs and plasma DHA levels; however, these findings were not consistent across all parameters and were not supported by multivariate analysis, so they should be interpreted with caution. Notably, a statistically significant association observed was a moderate positive correlation between plasma DHA concentrations and dietary ALA intake. However, this relationship did not persist in regression analysis, where neither dietary ALA intake nor the dietary n-6/n-3 PUFA ratio independently predicted plasma DHA levels. These findings suggest that reduced DHA status in MSUD patients cannot be explained by dietary intake patterns alone and may reflect underlying disturbances in fatty acid metabolism.

Research on LC-PUFA status in IMDs requiring a protein-restricted diet has predominantly focused on PKU. These studies have included all age groups, such as breastfed infants, children, adolescents and adults, and have measured PUFA levels in plasma and/or erythrocyte membranes [14, 22]. While findings regarding levels of specific fatty acids such as AA, LA and ALA have varied across studies [13, 14, 22], a consistent and prominent observation was the DHA deficiency in individuals with PKU [13, 14, 22, 23]. This DHA deficiency was initially attributed to insufficient dietary intake of DHA due to the avoidance of animal-derived foods [14, 23]. However, several subsequent studies failed to show a direct correlation between dietary intake and DHA status [11]. Some also reported higher erythrocyte DHA concentrations in individuals with better metabolic control and lower plasma phenylalanine levels [24], suggesting that factors beyond intake – such as impaired endogenous synthesis or disrupted fatty acid metabolism – may also contribute. Intervention studies examining the effects of LC-PUFA supplementation, particularly DHA have demonstrated that this deficiency can be corrected both in infancy and later in life [13, 14, 16, 25]. While some studies have reported improvements in visual evoked potentials following DHA supplementation [15], others found no significant effects on neurocognitive or visual outcomes [25]. Reflecting the accumulated evidence, recent PKU guidelines underscore the importance of individualized nutritional strategies for LC-PUFA supplementation and specifically recommend that women with PKU who are planning a pregnancy or are pregnant should receive DHA supplementation to support optimal fetal development [26].

Beyond PKU, studies on fatty acid composition and related metabolic disturbances in other IMDs are limited and often constrained by small sample sizes and heterogeneous patient groups [19]. In one study of five patients with methylmalonic acidemia (MMA) and eight with urea cycle disorders (UCD), plasma and red blood cell fatty acid profiles showed significantly elevated LA levels, along with reductions in ALA, AA and DHA [18]. Similarly, another small study involving four MMA patients reported increased ALA levels but no significant differences in AA or DHA concentrations [27]. In a separate cohort of four patients with propionic acidemia (PA), no significant changes were found in PUFA precursors (LA and ALA), intermediate metabolites (GLA and EPA), or end-products (AA and DHA) [28]. Additionally, a mixed-cohort study including patients with UCD, organic acidemias (MMA, PA, MSUD), tyrosinemia type 1, and homocystinuria reported elevated LA levels and reduced ALA, AA, and DHA; however, the absence of subgroup-specific analyses limits the ability to draw definitive conclusions about disease-specific PUFA metabolism [17]. Overall, these findings suggest that disturbances in LC-PUFA profiles may be common across various IMDs, but the evidence remains fragmentary and further targeted investigation is needed.

The first study in the literature to investigate fatty acid profiles in MSUD patients was conducted with six female patients aged 12 to 30 years and analyzed plasma and erythrocyte fatty acid compositions, including DHA, ALA, LA, EPA and AA [20]. The main finding was a significant reduction in DHA levels in both plasma and erythrocytes, despite elevated ALA concentrations. This suggests an impairment in the endogenous conversion of ALA to DHA, potentially related to altered fatty acid metabolism in MSUD. Dietary records confirmed minimal or absent DHA intake and the type of medical formula consumed did not significantly affect fatty acid status, highlighting the possible need for targeted LC-PUFA supplementation in this patient group [20]. In developing and testing a specialized formula for MSUD patients to optimize amino acid transport to the brain and compensate for nutrient deficiencies, Strauss et al. identified significant n-3 PUFA deficiencies associated with the standard medical nutrition formula [21]. Docosahexaenoic acid was found at less than 50% of normal levels in patient red blood cell membranes. In response, a study formula was specifically designed to address these deficiencies and was enriched with ALA. While the study formula increased membrane n-3 PUFA content and reduced the n-6 to n-3 PUFA ratio– suggesting a more physiologic balance among long-chain functional lipids– it failed to normalize DHA levels. Notably, the ratio of DHA to its precursor, docosapentaenoic acid (DPA; 22:5n-3), was decreased twofold at baseline and threefold during use of the study formula, indicating a potential functional block at the final step of n-3 PUFA synthesis [21]. Consistent with previous reports, our findings also demonstrated a significant reduction in plasma DHA concentrations in patients with MSUD. Regarding the mechanisms underlying DHA deficiency in MSUD patients, correlation analysis identified a moderate positive relationship between plasma DHA concentrations and dietary ALA intake. However, this association did not persist when dietary variables were evaluated simultaneously, as multivariate regression analysis showed that dietary ALA intake and the n-6/n-3 PUFA ratio did not independently influence plasma DHA levels. Taken together, these findings indicate that the observed DHA deficiency in MSUD patients may not be solely attributable to insufficient dietary intake but may also reflect an underlying impairment in LC-PUFA metabolism– possibly due to enzymatic dysfunction or disease-specific metabolic alterations.

Another strength of our study is the inclusion of MA in the fatty acid panel. Mead acid, an n-9 PUFA, is a well-established biomarker of essential fatty acid deficiency. It is endogenously synthesized from oleic acid via a pathway involving the genes *FADS2, ELOVL5* and *FADS1*, particularly under conditions of low dietary intake of n-3 and n-6 essential fatty acids such as LA and ALA. In states of essential fatty acid deficiency, decreased synthesis of n-6 and n-3 PUFAs results in a compensatory upregulation of MA production [29, 30]. Another important indicator of essential fatty acid deficiency is the ratio of mead acid to arachidonic acid, commonly referred to as the triene/tetraene ratio or Holman Index. The Holman Index, defined as the plasma MA/AA ratio, has historically been used for the biochemical diagnosis of EFAD, with a diagnostic threshold value of ≥0.20. However, it has been suggested that optimal cut-off values may vary across different age groups and clinical risk populations and should therefore be interpreted within the appropriate clinical and metabolic context [31].

In our study, MSUD patients had significantly higher plasma levels of MA. However, the observed elevation of MA in our cohort suggests a regulatory shift in essential fatty acid metabolism rather than classical essential fatty acid deficiency. Unlike the biochemical pattern typically seen in essential fatty acid deficiency, LA levels were preserved, and the MA/AA ratio was not significantly elevated. Notably, although oleic acid is the dietary precursor of n-9 fatty acids, dietary oleic acid intake was significantly lower in MSUD patients compared to controls, while plasma MA levels were higher in the patient. This inverse pattern further supports that elevated MA levels are unlikely to be explained solely by dietary n-9 fatty acid intake. These findings indicate that MA elevation in MSUD patients may reflect metabolic adaptations, such as altered desaturase and elongase activity, changes in fatty acid flux between lipid compartments, or broader metabolic remodeling affecting circulating fatty acid profiles, rather than an overt deficiency of essential fatty acids. Importantly, these interpretations should be considered hypothesis-generating, as the present study did not include functional assessments of enzyme activity, tissue-specific fatty acid measurements, or compartmental analyses of esterified and non-esterified fatty acid fractions. Therefore, further functional and mechanistic studies are needed to clarify the biological basis of MA elevation and its clinical relevance in MSUD.

This study has several limitations. First, fatty acid profiling was performed only in plasma samples rather than in erythrocyte membrane phospholipids, which may not fully reflect long-term fatty acid status. Although plasma PUFA measurements are widely used, practical, and analytically standardized, they are more susceptible to short-term metabolic and dietary fluctuations than erythrocyte-based assessments. To minimize this variability, all samples were collected during metabolically stable periods, and conditions known to significantly affect plasma fatty acid levels – such as acute illness, catabolic states, or metabolic decompensation – were carefully excluded through concurrent clinical and biochemical evaluation. Additionally esterified and non-esterified fatty acid fractions were not distinguished, which may represent a potential metabolic confounder. Second, the study was observational and did not include any intervention or supplementation, such as DHA or LC-PUFA-enriched medical foods. Therefore, we could not assess whether the observed deficiencies could be corrected through targeted dietary strategies. Another limitation of this study is that dietary AA intake could not be assessed. This limits the ability to distinguish between dietary and endogenous contributions to plasma AA levels. An additional limitation of this study is the relatively small sample size, which may have limited the statistical power of the analyses. As a result, some correlation and regression models may not have detected significant associations despite moderate effect sizes. Therefore, the absence of statistically significant predictors in multivariate analyses should be interpreted with caution, and larger studies are needed to further clarify the relationships between dietary intake, fatty acid metabolism, and plasma DHA status in MSUD. Finally, although DHA is known to play a critical role in retinal and cognitive function, our study did not include concurrent neurocognitive testing, electrophysiological evaluations or visual assessments.

In conclusion, this study provides evidence of DHA deficiency in patients with MSUD, along with changes in long-chain PUFA profiles. Although insufficient dietary intake may contribute to reduced DHA status, our findings indicate that additional mechanisms beyond intake alone may be involved and require further investigation. An important consideration for future research is the distinction between infants and older patients, as endogenous conversion of DHA from ALA is insufficient to meet the high demands of brain development in early life, emphasizing the need for age-specific evaluations of DHA status and requirements in MSUD. Given the observational nature of this study, the potential role of DHA supplementation cannot be determined from the current data and should be assessed in well-designed prospective and interventional studies.

## Supplementary information

Supplementary Material S1

Supplementary Material S2

Supplementary Material S3

Supplementary Material S4

## Data Availability

The data that support the findings of this report are available from the corresponding author upon reasonable request.
