# Supplementary material for: Evidence of docosahexaenoic acid deficiency in maple syrup urine disease: insights from plasma long-chain polyunsaturated fatty acid status
Source: Eur J Clin Nutr. 2026 Jun 12;80(9):895–903. doi: 10.1038/s41430-026-01774-7 (PMC13558074; doi:10.1038/s41430-026-01774-7)
Supplement: Supplementary file 1 [file 41430_2026_1774_MOESM1_ESM.docx]

**Supplementary Material S1: Method validation parameters for selected omega fatty acids**

| **Analyte** | **LOQ**  **(mg/L)** | **Linearity**  **(mg/L)** | **Recovery** | | **Repeatability** | | | |
| --- | --- | --- | --- | --- | --- | --- | --- | --- |
|  |  |  |  |  | **intra-day** | | **inter-day** | |
|  |  |  | **LLQC**  **(%)** | **HLQC**  **(%)** | **LLQC (%CV)** | **HLQC (%CV)** | **LLQC (%CV)** | **HLQC (%CV)** |
| Docosahexaenoic acid | 9.57 | 10.0 - 100.0 | 97 | 98 | 2.65 | 2.74 | 3.67 | 3.48 |
| Eicosatrienoic acid | 5.19 | 5.0 - 100.0 | 107 | 106 | 1.30 | 1.18 | 4.09 | 2.75 |
| Arachidonic acid | 8.5 | 15.0 - 300.0 | 98 | 98 | 3.31 | 3.15 | 8.80 | 7.24 |
| Eicosapentaenoic acid | 2.26 | 2.0 - 40.0 | 96 | 97 | 4.97 | 1.09 | 8.13 | 7.19 |
| Alpha-linolenic acid | 1.4 | 1.5 - 30.0 | 110 | 105 | 6.08 | 1.96 | 7.81 | 6.86 |
| Linoleic acid | 3.14 | 60.0 - 1200.0 | 98 | 98 | 7.01 | 1.01 | 8.21 | 4.01 |
| Gamma-linolenic acid | 3.35 | 4.0 - 80.0 | 99 | 100 | 7.74 | 3.13 | 8.69 | 7.98 |

The proposed method was validated according to the Q2(R1) ICH guidelines and provided satisfying results of accuracy, precision and other validation parameters.

*LOQ, Limit of Quantification; LLQC, Low-level quality control; HLQC, High-level quality control*
