# Supplementary material for: Evidence of docosahexaenoic acid deficiency in maple syrup urine disease: insights from plasma long-chain polyunsaturated fatty acid status
Source: Eur J Clin Nutr. 2026 Jun 12;80(9):895–903. doi: 10.1038/s41430-026-01774-7 (PMC13558074; doi:10.1038/s41430-026-01774-7)
Supplement: Supplementary file 2 [file 41430_2026_1774_MOESM2_ESM.docx]

**Supplementary Material S2: The demographic, clinical and molecular findings of the MSUD patients**

| **Patient** | **Sex** | **Gene** | **Nucleotide change^**^** | **Protein change** | **Age of diagnosis (days)** | **Current age (years)** |
| --- | --- | --- | --- | --- | --- | --- |
| 1 | Female | *BCKDHB* | c.93_103del | p. (Ala32Phefs*48) | 3 | 13 |
| 2 | Male | *na^*^* | na | na | 60 | 13 |
| 3 | Male | *BCKDHA* | c.773_774delinsAA | p.(Cys258*) | 7 | 9 |
| 4 | Male | *BCKDHB* | c.633+1G>T |  | 17 | 8 |
| 5 | Female | *na^*^* | na | na | 1295 | 6 |
| 6 | Female | *BCKDHB* | c.169C>T | p.(Gln57*) | 12 | 9 |
| 7 | Male | *BCKDHB* | c.1149T>A | p.(Tyr383*) | 6 | 6 |
| 8 | Male | *BCKDHB* | c.1149T>A | p.(Tyr383*) | 9 | 9 |
| 9 | Male | *BCKDHA* | c. 452C>T | p.(Thr151Met) | 343 | 17 |
| 10 | Female | *BCKDHA* | c.773_774delinsAA | p.(Cys258*) | 15 | 9 |
| 11 | Male | *BCKDHA* | c.773_774delinsAA | p.(Cys258*) | 23 | 7 |
| 12 | Male | *BCKDHB* | c.752T>C | p.(Val251Ala) | 7 | 24 |
| 13 | Female | *BCKDHB* | c.93_103del | p. (Ala32Phefs*48) | 7 | 20 |
| 14 | Female | *DBT* | c.1150A>G | p. (Ser384Gly) | 13 | 19 |
| 15 | Female | *na^*^* | na | na | 5 | 24 |
| 16 | Female | *BCKDHB* | c. 768C>G | p.(Tyr256*) | 20 | 18 |

* Diagnosis of MSUD confirmed by biochemical findings, including markedly elevated plasma leucine, valine, isoleucine and

alloisoleucine levels in quantitative amino acid analysis

** Reported variants represent the disease-causing genotype for each patient and are indicated as homozygous.

*na, not applicable*
