# Supplementary material for: Evidence of docosahexaenoic acid deficiency in maple syrup urine disease: insights from plasma long-chain polyunsaturated fatty acid status
Source: Eur J Clin Nutr. 2026 Jun 12;80(9):895–903. doi: 10.1038/s41430-026-01774-7 (PMC13558074; doi:10.1038/s41430-026-01774-7)
Supplement: Supplementary file 3 [file 41430_2026_1774_MOESM3_ESM.docx]

**Supplementary Material S3: Data on the macronutrient composition of the medical foods used in the study.**

| ***BCAA-free medical formula 1**** | | |
| --- | --- | --- |
| **Nutrients** | **Unit** | **Per 100g** |
| **Energy** | kcal | 74 |
| **Energy** | kj | 1260 |
| **Fat** | g | 0.20 |
| *Saturates* | g | 0 |
| **Carbohydrate** | g | 13.7 |
| *Sugars* | g | 1.0 |
| **Protein equivalent** | g | 60 |

*Used by patients 2,3,5,6,8,12

| ***BCAA-free medical formula 2**** | | |
| --- | --- | --- |
| **Nutrients** | **Unit** | **Per 100g** |
| **Energy** | kcal | 296 |
| **Energy** | kj | 1239 |
| **Fat** | g | 0 |
| **Carbohydrate** | g | 4.1 |
| *Sugars* | g | 0.2 |
| **Protein equivalent** | g | 70 |

*Used by patients 1,13,14,15,16

| ***BCAA-free medical formula 3**** | | |
| --- | --- | --- |
| **Nutrients** | **Unit** | **Per 100g** |
| **Energy** | kcal | 291 |
| **Energy** | kj | 1218 |
| **Fat** | g | 0 |
| **Carbohydrate** | g | 12.8 |
| *Sugars* | g | 0.42 |
| **Protein equivalent** | g | 60 |

*Used by patients 7,9

| ***BCAA-free medical formula 4**** | | |
| --- | --- | --- |
| **Nutrients** | **Unit** | **Per 100g** |
| **Energy** | kcal | 291 |
| **Energy** | kj | 1238 |
| **Fat** | g | <0.1 |
| **Carbohydrate** | g | 10.7 |
| *Maltodextrin* | g | 10.7 |
| **Protein equivalent** | g | 62.1 |

*Used by patients 4,10

| ***BCAA-free medical formula 5**** | | |
| --- | --- | --- |
| **Nutrients** | **Unit** | **Per 100g** |
| **Energy** | kcal | 339 |
| **Energy** | kj | 1440 |
| **Fat** | g | 0.05 |
| *Saturates* | g | 0 |
| **Carbohydrate** | g | 42.9 |
| *Sugars* | g | 27.1 |
| **Protein equivalent** | g | 41.7 |

*Used by patients 11

| ***Low protein medical product 1**** | | |
| --- | --- | --- |
| **Nutrients** | **Unit** | **Per 100g** |
| **Energy** | kcal | 528 |
| **Energy** | kj | 2210 |
| **Fat^**^** | g | 29.9 |
| *Saturates* | g | 12.57 |
| *Monounsaturates* | g | 12.2 |
| *Polyunsaturates* | g | 5.12 |
| **Carbohydrate** | g | 59.5 |
| *Sugars* | g | 58 |
| **Protein equivalent** | g | 4.5 |

*Used by patients 4,5,10,11,14

** Specific n-3 and n-6 fatty acid content could not be obtained from manufacturer data and therefore is not reported.

| ***Low protein medical product 2**** | | |
| --- | --- | --- |
| **Nutrients** | **Unit** | **Per 100g** |
| **Energy** | kcal | 536 |
| **Energy** | kj | 2242 |
| **Fat** | g | 32 |
| *Saturates* | g | 13.2 |
| *Monounsaturates* | g | 12.3 |
| *Polyunsaturates* | g | 5.8 |
| **Linoleic acid** | g | 5.2 |
| Alpha-linolenic acid | g | 10.3 |
| Docosahexaenoic acid | g | 0 |
| Arachidonic acid | g | 0 |
| **Carbohydrate** | g | 62 |
| *Sugars* | g | 2.4 |
| **Protein equivalent** | g | 0 |

*Used by patients 7,9
