# Supplementary material for: Evidence of docosahexaenoic acid deficiency in maple syrup urine disease: insights from plasma long-chain polyunsaturated fatty acid status
Source: Eur J Clin Nutr. 2026 Jun 12;80(9):895–903. doi: 10.1038/s41430-026-01774-7 (PMC13558074; doi:10.1038/s41430-026-01774-7)
Supplement: Supplementary file 4 [file 41430_2026_1774_MOESM4_ESM.docx]

**Supplementary Material S4: Correlation analysis between dietary fatty acid intake variables and plasma DHA concentration.**

| **Parameters** |  | **Plasma DHA level** | **Age** | **Dietary n-6/n-3 PUFA ratio** | **Dietary ALA intake** | **Dietary n-3 PUFA intake** |
| --- | --- | --- | --- | --- | --- | --- |
| **Plasma DHA level** | r  p | **-** | -0.015   0.957 | -0.225   0.402 | **0.516   0.041** | 0.497  0.05 |
| **Age** | r  p | -0.015   0.957 | **-** | 0.434 0.093 | -0.182  0.500 | -0.206  0.444 |
| **Dietary n-6/n-3 PUFA ratio** | r  p | -0.225   0.402 | 0.434  0.093 | **-** | -0.419  0.107 | -0.497  0.05 |
| **Dietary ALA intake** | r  p | **0.516  0.041** | -0.182 0.500 | -0.419  0.107 | **-** | **0.988  <0.001** |
| **Dietary n-3 PUFA intake** | r  p | 0.497  0.05 | -0.206  0.444 | -0.497  0.05 | **0.988  <0.001** | **-** |

Spearman Correlation (<0.25 very weak correlation; 0.26-0.49 weak correlation; 0.50-0.69 medium correlation; 0.70-0.89 high correlation; 0.90-1.0 very high correlation)

*PUFA, polyunsaturated fatty acid; DHA, docosahexaenoic acid; ALA, alpha-linolenic acid*
